# Factors associated with the absence of Brazilians in specialized dental centers

**DOI:** 10.1186/s12903-022-02402-z

**Published:** 2022-08-26

**Authors:** Inara Pereira da Cunha, Valéria Rodrigues de Lacerda, Gabriela da Silveira Gaspar, Edson Hilan Gomes de Lucena, Fábio Luiz Mialhe, Paulo Sávio Angeiras de Goes, Hazelelponi Querã Naumann Cerqueira Leite, Rafael Aiello Bomfim

**Affiliations:** 1 Public Health School Dr. Jorge David Nasser, Av. Sen. Filinto Müler, 1480 - Pioneiros, Campo Grande, MS 79074-460 Brazil; 2 Federal University of Mato Grosso Do Sul, Campo Grande, MS Brazil; 3 Federal University of Pernambuco, João Pessoa, PB Brazil; 4 Federal University of Paraíba, João Pessoa, PB Brazil; 5 Piracicaba Dental School, University of Campinas, Piracicaba, SP Brazil

**Keywords:** Absenteeism, Oral health, Unified health system, Secondary health care, Services use

## Abstract

**Aim:**

To identify the individual and contextual factors associated with the absence of Brazilians at a scheduled appointment in Dental Specialties Centers (DSC).

**Methods:**

This cross-sectional design uses the National Program for Improving Access and Quality of Dental Specialties Centers database, 2018. The outcome was the users' lack of at least one of the scheduled appointments. Contextual and individual independent variables were used, considering Andersen's behavioural model. The analyses were performed with the R Core Team and SAS (Studio 3.8, Institute Inc, North Carolina, U.S, 2019) programs.

**Results:**

Of the 10,391 patients interviewed, 27.7% missed at least one of the consultations. In the adjusted multivariate model, the interpretation based on the effect size and 95% CI showed that the behaviour individual predisposing factors such as age ≤ 42 years (OR = 1.10; 95%CI:1.01–1.21), individual need factors such as participation in the “Bolsa Família” program (OR = 1,14; 95%CI:1.02–1.27), not being covered by the Family Health Strategy (OR = 1.15; 95% CI:1.02–1.30), and users of periodontics services (OR = 1.22;95%CI:1.05–1.40) were associated with absences. The behavioural factor associated with the outcome was that the DSC facilities were not in good condition (OR = 1.18; 95%CI:1.03–1.34). DSC located in the capital (OR = 1.12; 95% CI: 0.92–1.48) were 12% more likely to have dental absences than those in the interior region.

**Conclusion:**

There are individual and contextual barriers associated with patients not attending specialised public dental consultations. DSC should offer adequate hours to patients, especially young adults and vulnerable people.

## Introduction

Failure to attend previously scheduled health appointments without the patient's justification is classified as absenteeism or interrupted consultations [1]. Worldwide, 23% of consultations scheduled for health treatments are not performed [2]. This behaviour can result from several social barriers [2] and even from the organisational structure of health institutions [3, 4]. A series of studies have shown that patients' non-attendance, especially to dental consultations, is associated with low income [4–6], less education [5], gender [5, 7], location and distance from services [6], waiting time for treatment [4] as well as their financial costs [8]. Despite advances in understanding this phenomenon, research on absenteeism in specialised public health services is scarce [9, 10]. However, such studies are necessary for elucidating associated factors with absenteeism. Indeed, it causes damage to the patients themselves, worsens oral problems, interrupts the workflow, and can reduce other patients' access to treatments and oral health care [11]. Still, the lack of patient care at the scheduled time leads to the underutilisation of the structural capacity of the public health system, generating financial costs and waste of human resources [12].

In Brazil, patients who seek dental services can be seen in the Unified Health System (UHS), by Dentists, from Primary Health Care (PHC). These professionals are inserted in the Family Health Strategy (FHS), Basic Health Units (BHU), or Emergency Care Units. FHS is the predominant PHC modality in the UHS, reaching 62.6% of Brazilians in 2019 [13]. All patients treated at the PHC level, who need complex dental interventions, such as diagnosis of oral pathologies, specialized periodontics, minor oral surgery, endodontics and care for people with special needs, are referred to the Dental Specialities Centers (DSC). After completing the dental treatment at the DSC, these patients are referred to the PHC oral health team. After that, patients will be accompanied by these professionals in periodic consultations [14].

However, the referral and scheduling of users at the DSC do not guarantee the attendance of patients in specialised consultations [15]. Depending on the speciality, the DSC's waiting time for starting treatment can reach up to 30 days. Therefore, it discourages the patient from going to the scheduled appointments [16]. In addition, there are studies in the country that reveal specific difficulties of the population in adhering to dental treatment [17, 18].

As absenteeism is influenced by several factors [3], using a multifactorial theoretical model could assist in understanding this issue, supporting strategies to increase the presence of patients in public services. In this sense, the Andersen [19] behavioural model can be applied. The model considers that contextual and individual factors can mediate the use of health services and is a methodological structure that supports a better interpretation of the results considering predisposing, enablers, needs and behavioural factors [20].

Thus, this study hypothesizes that individual and contextual factors are associated with the absence of users in specialised dental consultations in the country's Unified Health System. Therefore, the present study aimed to analyse the factors associated with patients' absences in consultations at DSC.

## Methods

This is a cross-sectional study with secondary data obtained from the external evaluation of the second cycle of the National Program for Improving Access and Quality of Dental Specialties Centers, conducted by the Brazilian Ministry of Health in 2018.

The external evaluation of the National Program for Improving Access and Quality of Dental Specialties Centers constituted three of four program phases. In this phase, there is on-site verification of access and quality standards in health services [21]. The questionnaires of the National Program for Improving Access and Quality of Dental Specialties Centers were composed of three modules, all in questionnaire formats, which were applied, in person, by an evaluator not linked to the health services. The first module referred to an observation of the physical structure and dental supplies, the second was an interview with the manager and a Dentist from the DSC, and the third module consisted of an interview with users in the health establishment. In addition, all evaluators underwent training and calibration processes.

The data collected by the National Program for Improving Access and Quality of Dental Specialties Centers were exported from the website (https://aps.saude.gov.br/ape/pmaq/ciclo2ceo/) to the Microsoft Office Excel 2016 program (Redmond, USA). The modules II and III spreadsheets were connected using the merge procedure, using a standard variable related to health services. With this procedure, we could identify the DSC Manager and dentist's responses to health services connected with their respective patients.

The sample consisted of 1,042 DSC from all over Brazil. Ten thousand three hundred ninety-one interviews were conducted with users at the National Program for Improving Access and Quality of Dental Specialties Centers. The survey used age as the inclusion criteria. Therefore, only persons over 18 years were included. In addition, patients in the establishment for the first time were excluded.

### Outcome

The absence of a Brazilian at a scheduled appointment in specialised public dental services was considered the outcome. The question was: "When you interrupt the treatment for any reason or do not come to the consultation, the professionals seek you to find out what happened and return to the service?” The possible answers were: Yes, the Community Agent of the FHS/ Yes, the professionals of the DSC themselves / No / Never abandoned or missed. To dichotomise the variable, the lack of patients at least one of the specialised public dental consultations was considered when patients answered that some professional was in contact or that they had not been contacted. Patients who answered that they never abandoned or missed were categorised as not missing the dental appointments.

### Covariates

**Fig. 1:**
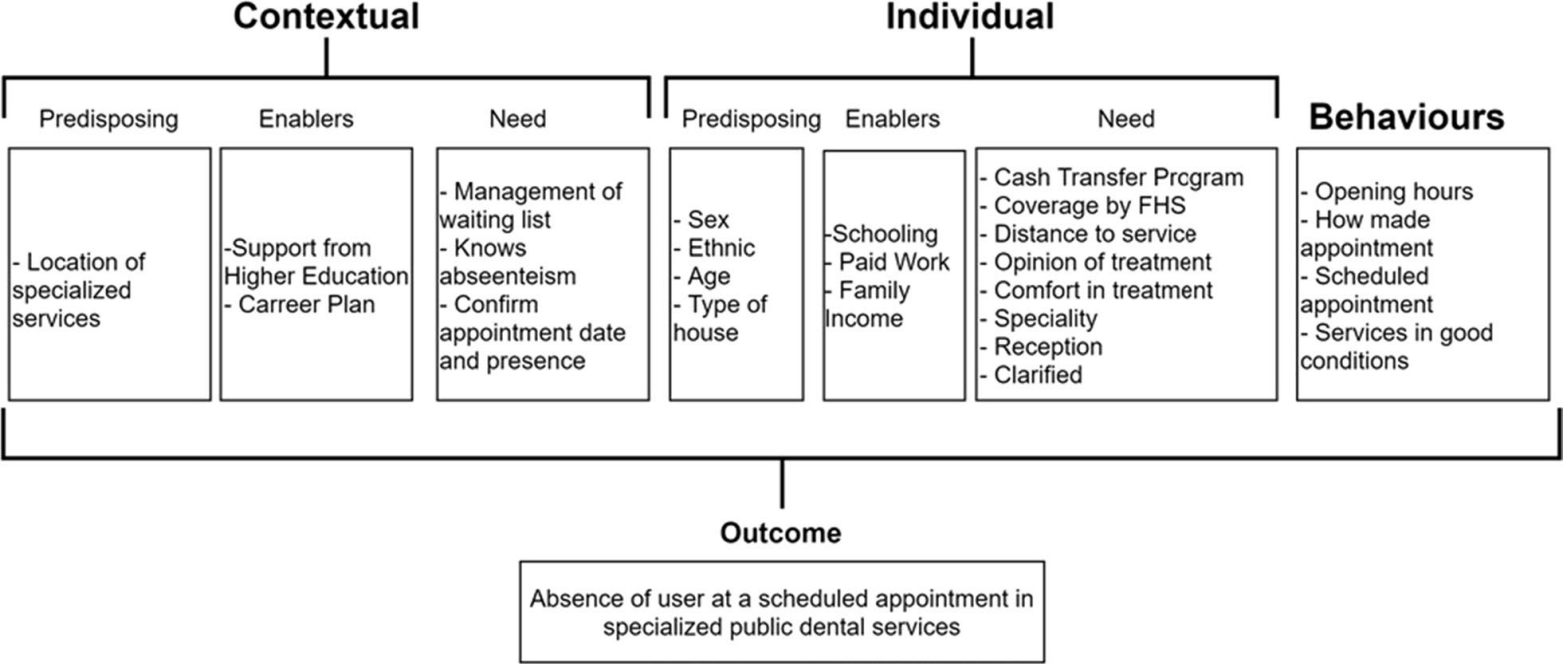
Contextual and individual variables of the study

The independent variables were grouped into two analysis components (contextual and individual), as shown in Fig. 1. For this, the behavioural model of Andersen [15] was used. The first component was composed of the contextual characteristics taken from module II of the National Program for Improving Access and Quality of Dental Specialties Centers. The location of the DSC (capital/interior city) was considered a predisposing contextual characteristic.

The following enabling contextual characteristics were analysed: the DSC receiving support from Higher Education Institutions to organise planning and work (yes / no), and the DSC's professionals having a career plan (yes/no). In addition, contextual characteristics of need were: to perform management of the waiting list (yes / no), the DSC to know the monthly percentage of patient absenteeism (yes/no), and the DSC to confirm the date and presence in the consultation (yes/no).

Regarding individual characteristics, the individual predisposing factors were sex, age (dichotomised by the mean ≤ 42 and > 42 years), race/colour (questioned according to the Brazilian Institute of Geography and Statistics, and dichotomised in whites and non-whites), and type of house (urban/rural). Enabling individual factors were considered schooling (dichotomised in ≤ high school and > high school), presence of paid work (yes / no), and family income (dichotomised by the median of ≤ 1 minimum wages and > 1 minimum wages).

Individual need factors were considered: participation in a cash transfer program known as the “Bolsa Família" program (yes / no), reporting coverage of the FHS (yes / no), mentioning the distance in minutes to reach the DSC (dichotomised by the average of ≤ 20 min and > 20 min). For the speciality, the question was considered: "What speciality is your treatment for?".

Individual behavioural factors were analysed and measured by the following questions: Does this DSC's opening hours meet your needs? (yes / no). How do you make an appointment with this DSC? (FHS marked/scheduled directly at the service/others). It was asked how the patient scheduled the appointment (With scheduled time/others / do not know). It was also assessed whether the patient considered the DSC in good condition (yes/no).

Frequency distribution tables were built. The associations between the absence of patients and the individual and contextual variables were performed using simple and multiple logistic regression models. Multilevel models were taken into account. In this sense, individuals were at the first level and DSC at the second level. The intraclass correlation coefficient was calculated from the model with only the intercept, estimating the proportion of the total variance due to the context (DSC). The measure of association used was the crude odds ratio (OR) and adjusted with the respective 95% confidence intervals. The variables selected for the multivariate model were based on statistical significance (*p* < 0.05) or effect sizes higher than 10%. Final interpretations were based on effect size estimates and 95% confidence intervals. The fit of the models was assessed by the QICC (Quasi-likelihood under the Independence model Criterion). The analyses were performed with the R Core Team (R Foundation for Statistical Computing, Vienna, Austria, 2020) and SAS (Studio 3.8, Institute Inc, North Carolina, U.S, 2019) programs.

The National Program for Improving Access and Quality of Dental Specialties Centers was carried out under the standards required by the Declaration of Helsinki and approved by the Research Ethics Council under protocol 23458213.0.1001.5208. In addition, all participants received and signed the Informed Consent Form in two copies.

## Results

The prevalence of patients who missed appointments was 27.7% (95% CI: 26.8% -28.5%). Table 1 presents the descriptive analysis of the patients' variables. It is observed that 67.5% were female, 66.4% with at least a high school education, 43.1% with paid work and 56.6% with an income of up to one minimum wage. It is also noted that 24.1% of patients receive a family allowance, 83.5% live in urban areas and 80.0% with FHS coverage. For 96.8% of the participants, the DSC's opening hours do not meet their needs. Still, 48.2% stated that consultations are by appointment. It is also observed that 92.4% answered that the surgeon-dentist explained the treatment during consultations.

**Table 1:** Distribution of frequencies of patients in the sample as a function of variables, n = 10.391

Variable	Frequency of patients (^1^%)	Frequency (^2^%) of absence in scheduled appointments in specialised public dental services
Absence of user at a scheduled appointment in specialised public dental services		
No	7516 (72.3)	–
Yes	2875 (27.7)	
Predisposing		
Sex		
Male	3376 (32.5)	901 (26.7)
Female	7015 (67.5)	1974 (28.1)
Age		
≤ 42 years	5294 (51.0)	1532 (28.9)
> 42 years	4670 (44.9)	1233 (26.4)
No information	427 (4.1)	110 (25.8)
Color /race		
White	3756 (36.2)	1026 (27.3)
Black	1376 (13.2)	407 (29.6)
Brown	4777 (46.0)	1327 (27.8)
Yellow/indigenous or ignored	482 (4.6)	115 (23.9)
Type of housing		
Urban	8676 (83.5)	2401 (27.7)
Rural	1649 (15.9)	458 (27.8)
No information	66 (0.6)	16 (24.2)
Facilitators		
Education		
Until incomplete high school	3489 (33.6)	950 (27.2)
At least complete high school	6902 (66.4)	1925 (27.9)
Paid work		
Yes	4475 (43.1)	1141 (25.5)
No	5681 (54.7)	1548 (27.2)
No information	235 (2.3)	186 (79.2)
Family income (Minimum wage)		
≤ 1	5885 (56.6)	1710 (29.1)
> 1	4506 (43.4)	1165 (25.8)
Need		
Participates in “Bolsa Família”		
Yes	2507 (24.1)	735 (29.3)
No	7726 (74.4)	2116 (27.4)
No information	158 (1.5)	24 (15.2)
FHS coverage		
Yes	8317 (80.0)	2259 (27.2)
No	1823 (17.5)	561 (30.8)
No information	251 (2.4)	55 (21.9)
Time to DSC		
Up to 20 min	6388 (61.5)	1749 (27.4)
More than 20 min	4003 (38.5)	1126 (28.1)
Service at the DSC		
Good	10,043 (96.6)	2770 (27.6)
Bad	39 (0.4)	15 (38.5)
Reasonable / Don't know/didn't answer /	309 (3.0)	90 (29.1)
Dentist clarified about treatment		
Yes	9606 (92.4)	2654 (27.6)
No	785 (7.6)	221 (28.2)
Speciality		
Periodontics	1782 (17.2)	536 (30.1)
Endodontics	2889 (27.8)	788 (27.3)
General surgery	2361 (22.7)	615 (26.0)
Others	2995 (28.8)	849 (28.4)
Do not know / Did not answer	364 (3.5)	87 (23.9)
Behaviours		
Opening hours meet the needs		
Yes	328 (3.2)	78 (23.8)
No	10,063 (96.8)	2797 (27.8)
How to schedule the consultation		
Receive the guide at FHS and book directly with the DSC	4570 (44.0)	1261 (27.6)
Others	5821 (56.0)	1614 (27.7)
Expected time to start treatment		
One week	3198 (30.8)	863 (27.0)
1 Month	3713 (35.7)	1053 (28.4)
More than one month	3480 (33.5)	959 (27.6)
Appointment for consultation		
By appointment	5007 (48.2)	1406 (28.1)
Others	5316 (51.2)	1447 (27.2)
Installation of the DSC in good condition		
Don't know / Didn't answer	68 (0.6)	22 (0.8)
Yes	8900 (85.6)	2415 (27.1)
No	1491 (14.4)	460 (30.8)

^1^ Percentage in the column; ^2^ Percentage of users in each category with missed appointments

Table 2 presents the results of the variables related to the DSC. It is noted that most of the sample were patients of DSC from municipalities in the interior (86.4%) who manage the waiting list (74.8%).

**Table 2:** Distribution of patients' frequencies as a function of contextual variables (from the Dental Specialities Centers—DSC), n = 10.391

Variable	Frequency of users evaluated (^1^%)	Frequency (^2^%) of absence in scheduled appointments in specialised public dental services
Predisposing		
DSC location		
Capital	1412 (13.6)	418 (29.6)
Interior	8979 (86.4)	2457 (27.4)
Facilitators		
DSC receives support from HEI		
Yes	1606 (15.5)	417 (26.0)
No	6593 (63.4)	1854 (28.1)
No information	2192 (21.1)	604 (27.6)
DSC professionals have		
Yes, All	3398 (32.7)	946 (27.8)
Some professionals	1710 (16.5)	499 (29.2)
No	5140 (49.5)	1387 (27.0)
No information	143 (1.4)	43 (1.5)
Need		
Manages waiting list		
Yes	7768 (74.8)	2165 (27.9)
No	2622 (25.2)	709 (27.0)
No information	1 (0.0)	1 (100.0)
The DSC knows the percentage of absenteeism		
Yes	7720 (74.3)	2115 (27.4)
No	2670 (25.7)	759 (28.4)
No information	1 (0.0%)	1 (100.0)
Actions to decrease absenteeism		
Prior confirmation of date and attendance	4848 (46.7)	1354 (27.9)
Others	2872 (27.6)	761 (26.5)
No information	2671 (25.7)	760 (28.4)

^1^ Percentage in the column; ^2^ Percentage of users in each category with missed appointments

Table 3 shows the results of the regression analyses. The intraclass correlation coefficient (ICC) was 0.177, indicating that the variability of patients' non-attendance at appointments between DSCs was 17.7%. The rest of the variability was due to variation between patients. Therefore, only the individual variables remained in the final model. The reduction in the typographical error of QICc of the final model compared to the empty model was 10.7%.In multivariate analysis, individual predisposition, facilitating, need and behavioural factors are associated with the absence of Brazilians at a scheduled appointment in specialised public dental services. It is observed that younger patients (OR = 1.10; 95% CI:1.01–1.21), who participate in the Bolsa Família program (OR = 1.14; 95% CI:1, 02–1.27), who live in regions without FHS coverage (OR = 1.15; 95% CI: 1.02–1.30), for consultations in the periodontics Specialty (OR = 1.22; 95% CI: 1.05–1.40) and who believe that the DSC's facilities are not in good condition (OR = 1.18; 95% CI: 1.03–1.34) were more likely to dental consultation absences. In addition, Dental Specialized Centers located in the capital (OR = 1.12; 95% CI: 0.92–1.48) were 12% more likely to have Dental absences than those in the interior region.

**Table 3:** Results of the Logistic Regression Models for the absence of Brazilians at a scheduled appointment in Dental Specialities Centers—DSC, n = 10.391

Variable	Crude analysis				Final multiple models			
	Estimate	SE	OR **Crude (IC95%)**	*p*-value	Estimate	SE	OR adjusted (IC95%)	*p*-value
Individual-level								
Predisposing								
Sex (Ref = M)	0.07	0.04	1.07 (0.98–1.17)	0.1058	–			
Age (Ref > 42)	0.10	0.05	1.10 (1.01–1.20)	0.0303	0.10	0.05	1.10 (1.01–1.21)	0.0384
Color/race black (Ref = White)	0.06	0.07	1.06 (0.92–1.22)	0.4254	–			
Color/race Browns (Ref = White)	0.04	0.05	1.04 (0.94–1.16)	0.4097	–			
House location (Ref = Urb)	0.01	0.06	1.01 (0.89–1.14)	0.9008	–			
Facilitators								
Education (Ref ≤ Until incomplete high school)	0.00	0.05	1.00 (0.91–1.10)	0.7710	**–**			
Paid work (Ref = Yes)	0.06	0.04	1.06 (0.97–1.16)	0.2109				
Family income (Ref > 1)	0.06	0.05	1.06 (0.97–1.17)	0.1845	–			
Need								
Participates in Bolsa Família (Ref = No)	0.10	0.05	1.11 (0.99–1.23)	0.0607	0.13	0.06	1.14 (1.02–1.27)	0.0216
FHS coverage (Ref = Yes)	0.12	0.06	1.13 (1.01–1.27)	0.0426	0.14	0.06	1.15 (1.02–1.30)	0.0261
Time to DSC (Ref ≤ 20 min)	0.03	0.04	1.03 (0.94–1.12)	0.5028	–			
Dentist clarified about treatment (Ref = Yes)	0.03	0.09	1.03 (0.87–1.23)	0.6995	–			
Specialty—Perio (Ref = surgery)	0.19	0.07	1.21 (1.06–1.39)	0.0056	0.20	0.07	1.22 (1.05–1.40)	0.0071
Specialty—Endo (Ref = surgery)	0.05	0.06	1.05 (0.93–1.19)	0.4168	0.07	0.06	1.08 (0.95–1.22)	0.2507
Specialty—Outras(Ref = surgery)	0.12	0.07	1.12 (0.98–1.28)	0.0813	0.12	0.07	1.13 (0.98–1.30)	0.0837
Behaviours								
Opening hours meet the needs (Ref = Yes)	0.11	0.12	1.11 (0.88–1.41)	0.3738	–	0.06	1.08 (0.82–1.40)	0.59
How to schedule the consultation (Ref = DSC)	− 0.04	0.05	0.96 (0.87–1.07)	0.5103	–			
Expected time to start treatment 1 Month (Ref = One week)	0.07	0.06	1.07 (0.95–1.20)	0.2567	–			
Expected time to start treatment is more than one month (Ref = 1 week)	0.05	0.06	1.05 (0.93–1.18)	0.4347	–			
Appointment for consultation (Ref = By appointment)	− 0.06	0.05	0.94 (0.85–1.05)	0.3020	–			
Installation of the DSC in good condition(Ref = Yes)	0.13	0.06	1.14 (1.01–1.29)	0.0436	0.16	0.07	1.18 (1.03–1.34)	0.0181
Contextual level (DSC)								
Predisposing								
DSC location (Ref = Interior)	0.12	0.09	1.13 (0.95–1.34)	0.1786	**–**	0.08	1.12 (0.92–1.48)	0.16
Facilitators								
DSC receives support from HEI (Ref = Yes)	0.10	0.09	1.10 (0.92–1.32)	0.3050	**–**	0.01	1.00 (0.98–1.02)	0.98
DSC professionals have career paths (Ref = Yes. All)	0.07	0.10	1.07 (0.88–1.31)	0.5056	**–**			
Need								
Manages waiting list (Ref = Yes)	− 0.04	0.08	0.96 (0.82–1.13)	0.6545	**–**			
The DSC Knows the percentage of absenteeism (Ref = Yes)	0.04	0.08	1.04 (0.89–1.22)	0.6330	**–**			
Actions to decrease absenteeism (Ref = Others)	0.07	0.08	1.08 (0.91–1.27)	0.3800	**–**			
QICc					10,943.96			

Variance between DSC = 0.1772; Residual variance = 0.8251; ICC = Intraclass correlation coefficient: Part of the total variation that is due to the contextual level (DSC) = 0.1768. QIC (empty model) = 12,259.13

*OR* Odds ratio, *CI* Confidence interval, *Estimate* model coefficient, *SE* Standard error of the model coefficient, *QICc* Quasi-likelihood under the Independence model Criterion

## Discussion

This study highlights two important findings. Firstly, individual predisposing factors, needs, and behaviours were associated with patients' absences in Dental Specialized Centers in Brazil. Secondly, those services in the capitals (more affluent areas) were at higher risk for absences in dental consultations than their counterparts.

This study has strengths and limitations. As this is a cross-sectional study, it is not possible to make causal inferences between the associations found. It was also impossible to control patients' possible migration between different health services. Furthermore, the question used to measure patients' absences is not specific about absenteeism, but it is believed that its adaptation has allowed it to have sufficient sensitivity to detect patients with at least one missed appointment. It is unknown whether this may have affected the completion of treatment at the service. On the other hand, it is a representative survey for the entire national territory, identifying contextual and individual factors using an interpretive theoretical model, the Andersen´s model.

Following our results on the Dental Specialties Centers in Brazil, it reached 27,7% of absenteeism. In another year in Brazil, the lack of patients in specialised treatments in the UHS reached 38% and caused a monetary waste, accumulated in three years, around R$ 18 million [9]. Over two years in Spain, the lack of patients in specialised consultations reached 13%, with losses of around 3 million euros [10]. The budgetary impact is not the only problem with patient shortages in Brazil. The absence of patients, in practice, reduces access to specialised care and compromises the principle of UHS integrality. Comprehensiveness guarantees assistance at the three levels of health care, whether within the scope of health promotion, prevention and recovery [22]. Therefore, it is observed that understanding the causes of patients' absences is also a means of proposing measures that affect the principle of integrality.

Andersen's [19] behavioural model was used to understand possible factors associated with the absence of consultations. This model explains the individual use of health services and access, determined by predisposition, needs, facilitators and behaviours [16], at individual and contextual levels. The use of this model to understand that access to dental services can be seen in the study by Wosley et al. (2017) [23]. When evaluating the literature on access to dental services, the authors identified PHC in use with contextual variables, including the organisation of dental services. Individual factors were associated with absences from consultations, including predisposing factors like the patient's age. A previous study found that of the 6,428 consultations scheduled at the DSC, more than 2,000 consultations were not performed due to patient absences. Most of the absentees were young adults [7]. Age has already been reported in other studies as a condition that influences the patient absences in dental consultations [5]. Most young adults work and study and report difficulties taking time off work to comply with the commitment to go to the dental appointment [24, 25]. That is why one of the critical measures to be taken by the services is to offer alternative hours to patients.

Individual need factors were also identified as conditions associated with missing appointments. The patient's participation in a cash transfer program, known as the “Bolsa Família Program”, stood out among the variables. The results showed that these patients are more likely to miss dental appointments. The Bolsa Família Program was a Federal Government strategy created in 2003. It ended in 2021, whose objective was to alleviate poverty, as it provided a monthly financial incentive for extremely poor or low-income families—monthly per capita income around R$60 to R$120 per month, respectively. Transfers to families corresponded to monthly transfers consisting of a fixed and a variable amount, from R$ 77.00 to R$ 435.00 [26]. To be included in the program, the family should fulfil some requirements to carry out periodic consultations in Primary Health Care. However, dental appointments were not mandatory. Thus, inserted in primary health care, oral health teams did not always carry out specific health education actions for this public [27]. This could also contribute to the lack of these people in specialized dental consultations.

It is then noticed that there is a profile of absent patients, and it is possible to state that those in more vulnerable conditions are more likely to be absent during dental treatment. Understanding the vulnerable segment with a higher propensity to be absent is essential information to develop strategies articulated between primary health care and the DSC (specialised care) to guarantee treatment for this population. This information reflects the need for the FHS teams, inserted in PHC, to develop educational actions that stimulate the perception of the need for treatment in this group [25] and monitor the people referred to secondary care.

The FHS coordinates and executes the attributes of PHC in Brazil. Thus, it is plausible that the individual need factor related to FHS coverage is associated with patients' absences. Failure to cover the FHS makes it difficult for the population to access specialised services [28]. It can also impair periodic and educational monitoring, affecting the development of the treatment needs among the most vulnerable [27], despite the increase in PHC coverage over the years. This reveals the need to expand primary health care coverage to guarantee full access to dental care in public services.

Still, as an individual need factor, it was observed that the speciality of periodontics was associated with the absence of consultations. In the country, the perception of the need for dental treatment is low [29], contributing to the lack of dental appointments for specialities. Furthermore, initial adherence to periodontal treatment is influenced by the patient's perception of the treatment of the disease and its functional impact on quality of life [30]. Thus, a probable low perception of periodontal need may have contributed to the findings. Also, financial difficulties will make it impossible to travel to the health service due to the lack of resources to pay for bus tickets.

Furthermore, dental treatments require returns, which would require several tickets [7]. It is expected from health units that, for this reason, treatments that require periodic returns, such as those related to the speciality of periodontics, allow the highest chance of absences among patients in the public dental service. In this sense, intersectoral public policies that facilitate and reduce displacement are necessary.

Failure to attend dental care is a complex behaviour mediated by several factors. Lapidos et al. [3] comment that adverse experiences in dental consultation could influence future dental appointments. A negative experience that affected the DSC's dental appointment attendance was identifying that the establishment was not in good condition. This was a behavioural factor and associated with patients' absences. The physical structure in proper conditions of use represents a characteristic of the quality of the service. It is as essential as reducing waiting time, training professionals, good professional-user relationships, cordial service and an adequate number of professionals [31, 32].

Furthermore, DSC located in the capital were associated with patient absences. Generally, capital DSC is a reference for other less affluent areas and cities located in the country's interior region. Therefore, it could be an associated factor in difficulties arriving at dental care in capital DSC.

Given the findings, dental care services must offer adequate hours to patients, especially young adults. Social assistance support to ensure access for the vulnerable population is essential. There is evidence that oral health conditions are precarious among beneficiaries of the “Bolsa Familia Program” in Brazil [33], reinforcing the need for access to dental treatment for the participants.

It is also necessary to consider the importance of professional guidance and services for consultations and returns during periodontal treatment. The expansion of the FHS coverage is an aspect that can assist in greater population access to specialised treatment since the more organised primary health care is, the better the flow of care at the secondary level. On the other hand, investments in the DSC's infrastructure can improve service quality and patient return. Thus, these notes should minimise patients' absences in consultations with specialised public services and guarantee comprehensive dental care. In conclusion, individual and contextual barriers are associated with patients not attending specialised public dental consultations. DSC should offer adequate hours to patients, especially young adults and vulnerable people.

## Data Availability

Datasets related to this article can be found on the Ministry of Health website hosted at https://aps.saude.gov.br/ape/pmaq/ciclo2ceo/
